# Neutrophilic Lung Inflammation Suppressed by Picroside II Is Associated with TGF-*β* Signaling

**DOI:** 10.1155/2015/897272

**Published:** 2015-11-05

**Authors:** Soohwan Noh, Kyung-Seop Ahn, Sei-Ryang Oh, Kyun Ha Kim, Myungsoo Joo

**Affiliations:** ^1^School of Korean Medicine, Pusan National University, Yangsan 626-870, Republic of Korea; ^2^Natural Medicine Research Center, Korea Research Institute of Bioscience and Biotechnology, Ochang, Chungbuk 363-883, Republic of Korea

## Abstract

Although acute lung injury (ALI) is a leading cause of death in intensive care unit, effective pharmacologic means to treat ALI patients are lacking. The rhizome of *Picrorhiza scrophulariiflora* used in a traditional herbal medicine in Asian countries has been shown to have anti-inflammatory function, and picroside II (PIC II) is known as a major constituent in the plant. Here, we examined whether PIC II has an anti-inflammatory activity, which is applicable for treating ALI. We found that although it is not significantly effective in suppressing proinflammatory factor NF-*κ*B or in activating anti-inflammatory factor Nrf2, PIC II induced the phosphorylation of Smad 2, with concomitant increase of luciferase activity from SBE luciferase reporter in RAW 264.7 cells. H&E staining of lung, differential counting of cells in bronchoalveolar lavage fluid, and semiquantitative RT-PCR analyses of lung tissues show that an intratracheal (i.t.) spraying of PIC II suppressed neutrophilic inflammation and the expression of proinflammatory cytokine genes in the lung, which were elicited by an i.t. LPS instillation to the lung. In addition, PIC II treatment increased the phosphorylation of Smad 2 in the lung tissue. Together, our results suggest that PIC II plays a role as an anti-inflammatory constituent in *P. scrophulariiflora*, whose activity is associated at least in part with TGF-*β* signaling.

## 1. Introduction

Acute lung injury (ALI) is a leading cause of death in critical care [1, 2]. Salient features of ALI include hypoxemia, diffused neutrophilic infiltration, and interstitial edema in the lung. Major cause of ALI is bacterial sepsis, and Gram-negative bacteria are frequent culprits [1]. Despite significant research and substantial efforts in developing treatments, ALI maintains a high mortality rate of roughly 40%, and no effective pharmacological treatment for the disease is available [1–3]. Therefore, developing a regimen or a drug effective against ALI is urgently needed.

The endotoxin of Gram-negative bacteria, lipopolysaccharide (LPS), plays a key role in eliciting lung inflammation by inducing the production of proinflammatory cytokines [4, 5]. LPS binds to Toll-like receptor 4 (TLR4), resulting in NF-*κ*B activation and thereby upregulating the expression of proinflammatory cytokines and chemokines such as TNF-*α*, IL-6, IL-1*β*, and IL-8 [4]. These cytokines and chemokines play critical roles in the development of ALI; they stimulate chemotaxis of leukocytes to the lung, increasing infiltration of inflammatory cells to the lungs of ALI/ARDS patients [6]. In addition, LPS induces the production of reactive oxygen species (ROS) [6]. ROS in turn activate nuclear erythroid 2-related factor 2 (Nrf2) [7], a key redox-sensitive transcription factor that regulates the expression of antioxidant, xenobiotic detoxification enzymes [8, 9]. Importantly, activation of Nrf2 is closely associated with suppressing lung inflammation in ALI and sepsis mouse models [10, 11]. Therefore, along with NF-*κ*B, Nrf2 is emerging as a new target for regulation of inflammation [12].

In self-limiting inflammatory responses, the level of transforming growth factor-*β* (TGF-*β*) in exudate is increased and plays an antiphlogistic function [13]. TGF-*β* mediates the egress of undifferentiated leukocytes, facilitating the resolution of inflammation and tissue repair [14]. TGF-*β* binds to and signals through the two TGF-*β* receptors (TGF-*β*RI and TGF-*β*RII) [15–17]. TGF-*β* binding to TGF-*β*RII subsequently recruits TGF-*β*RI to form a catalytically active TGF-*β* receptor [15], which then phosphorylates receptor-regulated Smads (R-Smads), Smads 1, 2, 3, 5, and 8, in cytoplasm [18]. The phosphorylated R-Smads move to the nucleus and bind to coactivator Smad 4 to form multisubunit complexes on Smad-binding element (SBE) in a cognate promoter, where the transcription of diverse genes starts, contributing to the suppression of inflammation [16, 19]. Smads are ubiquitously expressed in variety of cell types, among which Smad 2 and Smad 4 are known as canonical factors for transcriptional response to TGF-*β* [20].

The rhizome of* Picrorhiza scrophulariiflora* has been prescribed as part of Asian traditional medicine for the treatment of rather a broad range of diseases [21]. However, it was reported that the herb has immunomodulatory and anti-inflammatory functions. For instance, the ethanol extract of* P. scrophulariiflora* suppresses redox-sensitive inflammation [22], while the diethyl ether extract of* P. scrophulariiflora* reduces the classical pathway of complement activation, the production of ROS by activated neutrophils, and the proliferation of T lymphocytes [23]. Picroside II (PIC II) is known as a major constituent found in plant [24]. Therefore, in this study, we explored the possibility that PIC II has an anti-inflammatory activity which is effective for treating ALI. Using RAW 264.7 cells and an LPS-induced ALI mouse model, we show that PIC II was effective in suppressing neutrophilic lung inflammation and that the possible anti-inflammatory effect of PIC II was, at least in part, associated with TGF-beta signaling.

## 2. Materials and Methods

### 2.1. Reagents

All the chemicals including picroside II (PIC II) and sulforaphane (SFN) were purchased from Sigma Chemical Co. (St. Louis, MO, USA) unless specified otherwise. TLR4-specific LPS (*Escherichia coli* O55:B5) was purchased from Alexis Biochemical (San Diego, CA, USA). Murine TGF-*β*1 was purchased from Cell Signaling (Danvers, MA, USA). All antibodies used in this study were from Santa Cruz Biotechnology (Santa Cruz, CA, USA).

### 2.2. Animal

Male C57BL/6 mice were purchased from the Oriental Bio Korea, Ltd. (Seongnam, Korea) and inbred in a specific pathogen-free facility at Pusan National University, Yangsan, Korea. Animals were housed in certified, standard laboratory cages and fed with food and water* ad libitum* prior to experiment. All experimental procedures followed the guideline of NIH of Korea for the Care and Use of Laboratory Animals, and all the experiments were approved by the Institutional Animal Care and Use Committee of Pusan National University, Pusan, Korea (protocol number: PNU-2010-00028).

### 2.3. Animal Model for Acute Lung Injury and PIC II Administration

Mice were anesthetized by Zoletil (Virbac, Carros cedex, France) and received a single intratracheal (i.t.) spraying of 2 mg LPS (*Escherichia coli* O55:B5, Sigma, St. Louis, MO, USA)/kg body weight or sterile saline. LPS in 10 *μ*L of phosphate buffer saline (PBS) was loaded in MicroSprayer Aerosolizer (Penn-Century, Wyndmoor, PA, USA) and delivered in aerosol to the lung via trachea under visual guide. At 2 h after the intratracheal LPS administration, PIC II (0.5 or 1 mg/kg of body weight) in 10 *μ*L of phosphate buffer saline (PBS) was similarly loaded in the MicroSprayer Aerosolizer and delivered in aerosol to the lung via trachea. The dose of PIC II (0.5 mg/kg) was determined by approximating 10^−6^ M of PIC II (molecular weight, 512.46). At 24 h after intratracheal spraying of LPS, mice were euthanized by CO_2_ gas. The trachea was exposed through midline incision and cannulated with a sterile 24-gauge intravascular catheter. Bilateral bronchoalveolar lavage (BAL) was performed by two consecutive instillations of 1 mL of PBS. Total cell numbers in BAL fluid were counted with hemocytometer, and cells in BAL fluid were prepared by a cytospin and stained for the differentiation of macrophages, lymphocytes, or neutrophils by Hemacolor (Merck, Darmstadt, Germany). Three hundred cells in total were counted, and one hundred of the cells in each microscopic field were scored. The mean number of cells per field was reported. For collecting lung tissue, mice were perfused with saline and the whole lung was inflated with a fixative. After paraffin embedding, 5 *μ*m sections were cut and placed on charged slides and stained with hematoxylin and eosin (H&E) staining method. Three separate H&E-stained sections were evaluated in 100x microscopic magnifications per mouse.

### 2.4. Cell Culture

RAW 264.7 cells were obtained from ATCC (American Type Culture Collection, Rockville, MD, USA) and cultured in Dulbecco's Modified Eagle's Medium (DMEM) containing L-glutamine (200 mg/L) (HyClone, Logan, UT, USA) supplemented with 10% (v/v) heat-inactivated fetal bovine serum (FBS), 100 U/mL penicillin, and 100 *μ*g/mL streptomycin (Invitrogen, Carlsbad, CA, USA). The cells were cultured and maintained in a humidified incubator at 37°C and 5% CO_2_ prior to experiment.

### 2.5. Luciferase Assay

Cells were cotransfected with SBE luciferase reporter and constitutively active TGF-*β*R1 expressing plasmids (gifts from Dr. Kirk Lane at Vanderbilt University School of Medicine) along with* tk*-Renilla luciferase constructs. At 48 h after transfection, cells were treated with various amounts of PIC II for 16 h before cell harvest. Samples were triplicated, and experiment was performed three times independently. Luciferase assay was performed with a dual luciferase assay system and the manual of the manufacturer (Promega, Madison, WI). Renilla luciferase activity in each sample was used as the denominator for the firefly luciferase activity driven by SBE.

### 2.6. Tetrazolium-Based Colorimetric Assay (MTT)

MTT assay was performed to evaluate the cytotoxicity of PIC II on RAW 264.7 cells. Cells (1.0 × 10^4^ cells/well) were treated with PIC II for 16 h, to which MTT [3-(4,5-dimethylthiazol-2-yl)-2,5-diphenyltetrazolium bromide] solution (2.0 mg/mL) was added. After 4 h incubation at 37°C in a humidified incubator with 5% CO_2_, formazan crystals formed in viable cells were dissolved with DMSO and the optical density (OD) was measured at 540 nm. Samples were triplicated, and experiment was performed three times independently. Cell viability was calculated as a percentage against the untreated control.

### 2.7. Isolation of Total RNA from Cell and RT-PCR

Total RNA was isolated from lung tissue by using TRIzol reagent and the manufacturer's instructions (Invitrogen). After the concentration of RNA was determined by spectrophotometer, 2 *μ*g of RNA was reverse-transcribed by M-MLV reverse transcriptase (Promega, Madison, WI, USA). The quantity of each mRNA was determined by using end-point dilution PCR, including three serial 1 to 5 dilutions (1 : 1, 1 : 5, 1 : 25, and 1 : 125) of RT products prior to PCR amplification. To eliminate genomic DNA contamination, equal amounts of total RNA from each sample were amplified by PCR without RT reaction. A portion of the cDNA was amplified by PCR with a set of specific primers (Table 1). For PCR amplification, TaqPCRx DNA polymerase, Recombinant (Invitrogen), and the manufacturer's protocol were used. The reaction conditions were as follows: an initial denaturation at 95°C for 5 min followed by 25~35 cycles of denaturation for 30 sec at 95°C, annealing for 30 sec at 58°C (IL-1*β*), 60°C (IL-6), 58°C (TNF-*α*), or 57°C (GAPDH), and extension for 40 sec at 72°C with a final extension for 7 min at 72°C. Amplicons were separated in 1% Agarose gels in 1x boric acid buffer (100 mM boric acid, 150 mM NaCl, and pH 8) at 130 V for 15 min, stained with ethidium bromide, and visualized under UV light. Glyceraldehyde-3-phosphate dehydrogenase (GAPDH) was used as internal controls for evaluating relative expressions of IL-1*β*, IL-6, and TNF-*α*. Relative expression of each gene over GAPDH was determined by densitometric analysis software ImageJ (National Institute of Mental Health, Bethesda, Maryland, USA).

### 2.8. Western Blot Analysis

Total proteins and nuclear proteins from cells and lung tissues were extracted by using RIPA buffer and NE-PER nuclear extraction kit and the manufacturer's protocol, respectively (Thermo Scientific, IL, USA). The amounts of proteins were measured by Bradford (Bio-Rad, Hercules, CA, USA). Equal amounts of proteins were fractionated by SDS-PAGE and then transferred to PVDF membrane (Bio-Rad). Blots were blocked for at least 1 h with 5% nonfat dry milk prior to incubation with appropriate antibodies at 4°C overnight. After incubation with secondary antibodies conjugated with HRP for 1 h at room temperature, specific bands of interest were revealed by chemiluminescence (SuperSignal West Femto, Thermo Scientific).

### 2.9. Myeloperoxidase (MPO) Activity

MPO activity in lung homogenates of mice was determined by using the myeloperoxidase fluorometric detection kit and the manufacturer's instruction (Enzo Life Sciences International, Inc., New York, USA). Data were presented as unit/g tissue.

### 2.10. Statistical Analysis

Data are presented as mean ± SEM (standard error of the mean) of at least three separate experiments. For comparison among groups, paired or unpaired* t*-tests and one-way analysis of variance (ANOVA) tests were used (with the assistance of InStat, GraphPad Software, Inc., San Diego, CA). *P* values less than 0.05 were considered statistically significant.

## 3. Results

### 3.1. PIC II Was Not Effective in Suppressing NF-*κ*B Activity in RAW 264.7 Cells

**Figure 1:**
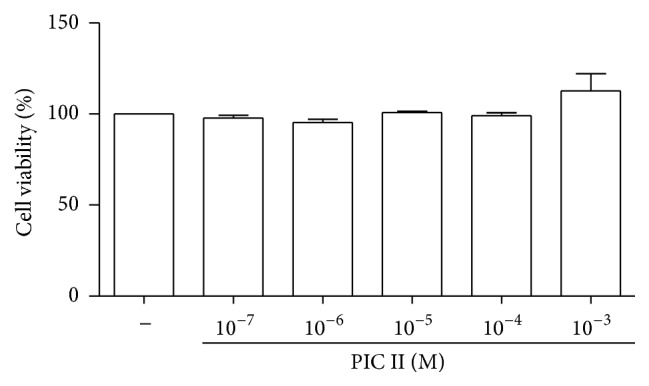
Effects of PIC II on cell viability. Cytotoxicity of PIC II was determined by MTT assay with RAW 264.7 cells. Results are representatives of at least three independent measurements. Data represent the mean ± SEM of triple sets.

For the determination of an optimal dose of PIC II with the least cellular toxicity, RAW 264.7 cells were treated with various amounts of PIC II, from 0.1 *μ*M to 1 mM, for 16 h, and the viability of the treated cells was measured by MTT assay. As shown in Figure 1, there was no significant cellular toxicity within the tested range of PIC II. For this study, we used PIC II from 0.1 *μ*M to 10 *μ*M.

**Figure 2:**
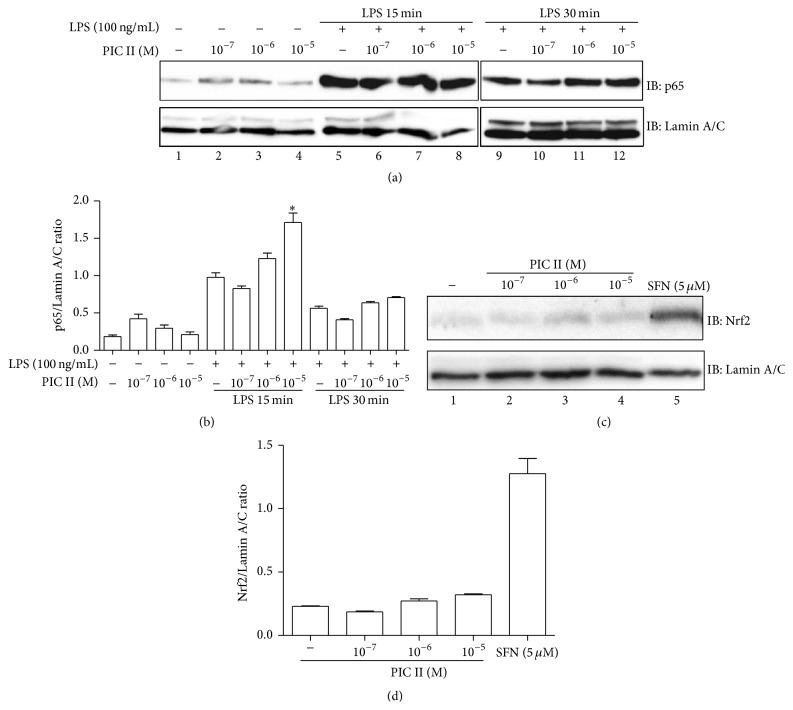
PIC II is not an effective suppressor of NF-*κ*B or activator of Nrf2. (a) RAW 264.7 cells were treated with indicated amounts of PIC II for 16 h and subsequently 15 min or 30 min with LPS. NF-*κ*B (p65 RelA) was measured by Western blot. The membrane was stripped and reprobed with *α*-Lamin A/C antibody for ensuring an equal loading of nuclear proteins. (b) The band intensity of each band was analyzed by ImageJ, and the relative intensity of p65 RelA was calculated over Lamin A/C. Results are representatives of at least three independent experiments. Data are shown as the mean ± SEM of three measurements. ^*∗*^ *P* was less than 0.05. (c) RAW 264.7 cells were treated with the indicated amounts of PIC II for 16 h along with sulforaphane (SFN, 4 h at 5 *μ*M) and Nrf2 was analyzed by Western blot. (d) Similar to (b), the relative intensity of Nrf2 was calculated over Lamin A/C by ImageJ. No statistical significance was found.

Next, we examined how PIC II exerts its anti-inflammation. First, given that NF-*κ*B is a key transcription factor that promotes inflammation [25], we tested whether PIC II exerts its anti-inflammatory function by suppressing NF-*κ*B activity (Figure 2). RAW 264.7 cells were treated with various amounts of PIC II for 16 h and then with LPS (0.1 *μ*g/mL) for 15 min or 30 min. Since activation of NF-*κ*B induces nuclear localization of p65 RelA, a subunit of NF-*κ*B [25], nuclear proteins were extracted and analyzed by Western blotting for nuclear p65 RelA. As shown in Figure 2(a), LPS treatment for 15 min induced nuclear localization of p65 RelA (lane 5), as opposed to the untreated control (lane 1). However, variable treatments with PIC II did not affect the localization of p65 RelA (lanes 6 to 7), although statistical analysis revealed that the treatment with 10^−5^ M of PIC II seemed to rather increase the localization of p65 RelA (lane 8 in Figure 2(a) and 8th column in Figure 2(b)). In similar experiment, while LPS treatment for 30 min prompted nuclear localization of p65 RelA (lane 9), treatments with PIC II did not significantly affect the localization of p65 RelA either (lanes 10 to 12 in Figure 2(a) and 10th to 12th columns in Figure 2(b)). These results suggest that PIC II is not effective in suppressing NF-*κ*B activity.

### 3.2. PIC II Was Not Effective in Activating Nrf2 in RAW 264.7 Cells

Since activation of Nrf2 is closely associated with suppression of inflammation [26], we tested whether PIC II exerts its anti-inflammatory function by activating Nrf2. RAW 264.7 cells were treated with various amounts of PIC II, along with sulforaphane (SFN), a pharmacologic activator of Nrf2 [27]. At 16 h after the treatments, cells were harvested, and nuclear proteins were isolated for Western blot analysis of Nrf2, indicative of activated Nrf2. As shown in Figures 2(c) and 2(d), PIC II did not significantly induce nuclear location of Nrf2, while SFN robustly activated Nrf2 (lane 5). Similarly, no induction of Nrf2-dependent genes by PIC II was detected by RT-PCR (data not shown), indicating that PIC II was not effective in activating Nrf2. Combined with the results of NF-*κ*B in Figure 2(a), these results suggest that the anti-inflammatory function of PIC II is not mediated by NF-*κ*B or Nrf2 in our experimental settings.

### 3.3. PIC II Induces Phosphorylation of Smad 2 in RAW 264.7 Cells

**Figure 3:**
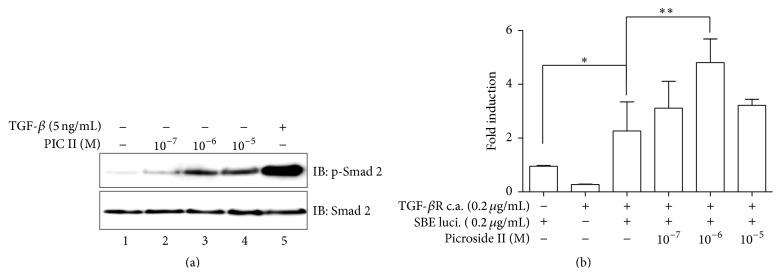
PIC II induces the phosphorylation of Smad 2 and enhances SBE-mediated transcriptional activity. (a) RAW 264.7 cells were treated with increasing amounts of PIC II. The phosphorylated form of Smad 2 (p-Smad 2) was measured by Western blot. The membrane was stripped and reprobed with Smad 2 for ensuring an equal loading of proteins. (b) RAW 264.7 cells were transfected with SBE luciferase reporter construct along with a plasmid encoding a constitutively active (c.a.) TGF-*β*R. Transfected cells were treated with indicated amounts of PIC II. Results are representatives of at least three independent experiments. Data were shown as the mean ± SEM of triple sets. ^*∗*^ *P* was less than 0.05, compared with reporter only, and ^*∗∗*^ *P* was less than 0.05, compared with the group transfected with the reporter and the c.a. TGF-*β*R.

Given that TGF-*β* is involved in suppressing inflammatory response [13], we tested the possibility that the anti-inflammatory activity of PIC II is associated with TGF-*β* signaling. As TGF-*β* signaling starts by active TGF*β*R phosphorylating Smad 2 [28], we tested whether PIC II induces the phosphorylation of Smad 2. RAW 264.7 cells were treated with various amounts of PIC II for 1 h, along with TGF-*β* (5 ng/mL) as a positive control. Total proteins were isolated from the variously treated cells and analyzed by Western blotting for the phosphorylated form of Smad 2. As shown in Figure 3(a), PIC II induced the phosphorylation of Smad 2 as low as 10^−7^ M (lane 2). The level of the phosphorylation of Smad 2 by PIC II was significantly increased at 10^−6^ M, albeit not as effective as TGF-*β*1. To test whether Smad 2 phosphorylated by PIC II is associated with increased transcriptional activity from a TGF-*β* dependent promoter, we transfected RAW 264.7 cells with SBE luciferase reporter construct that contains a Smad-binding site upstream of luciferase gene, along with a constitutively active TGF-*β*R1 expressing plasmid for 48 h. Transfected cells were treated with various amounts of PIC II for 16 h. As shown in Figure 3(b), PIC II treatment significantly increased the luciferase activity, suggesting that PIC II treatment enhances a transcription activity governed by TGF-*β* signaling. Together, these results suggest that PIC II is capable of phosphorylating Smad 2, a key factor in TGF-*β* signaling.

### 3.4. PIC II Suppresses Neutrophilic Lung Inflammation in LPS-Induced ALI Mice Model

**Figure 4:**
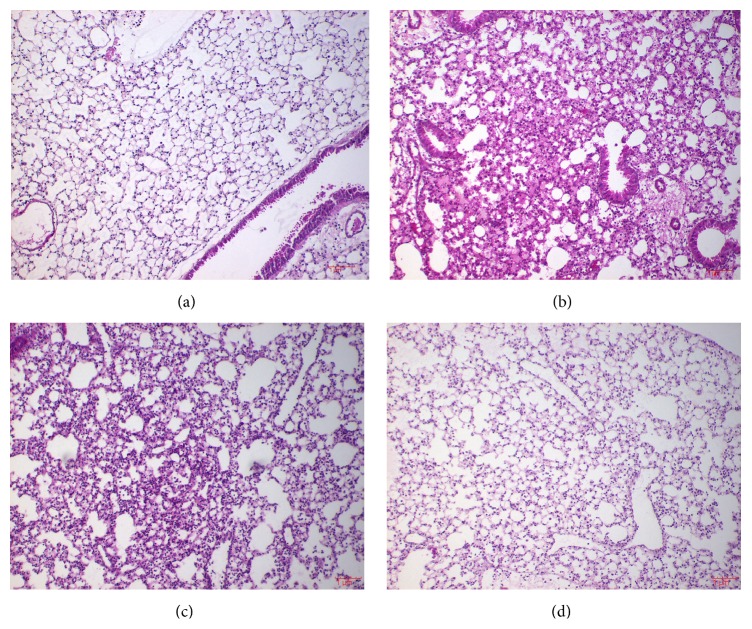
H&E staining of lung sections from LPS-induced ALI mice. ALI was induced in mice (*n* = 5/group). Mice received an intratracheal spraying of LPS to the lung (C57BL/6; (b), (c), and (d)) and subsequently two different amounts of PIC II [0.5 mg/kg body weight (c) or 1 mg/kg body weight (d)] or PBS (a). Representatives of at least five different areas of a lung (magnification 100x) are shown.

**Figure 5:**
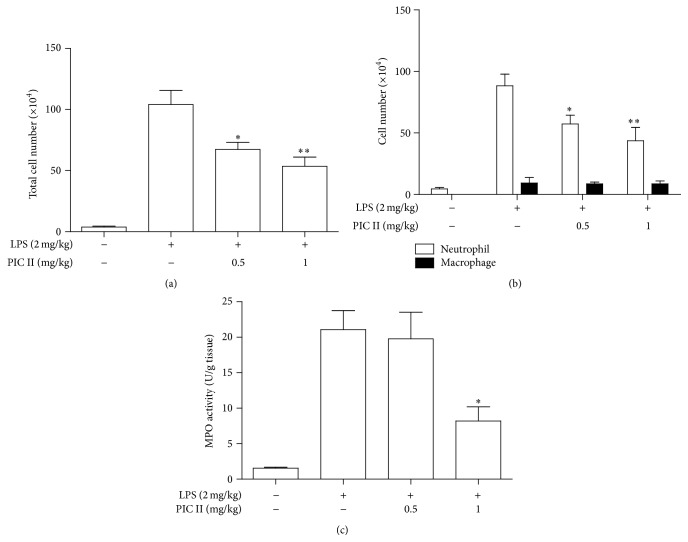
PIC II suppresses neutrophilic lung inflammation in ALI mice. Bronchoalveolar lavage (BAL) was performed with ALI mice (*n* = 5/group) treated with or without PIC II. Total cells in the lavage fluid (a) and neutrophils and macrophages (b) were counted. Data represent the mean ± SEM of 5 mice. ^*∗*^ *P* and ^*∗∗*^ *P* were less than 0.05 and 0.001, respectively, compared with the LPS only. (c) MPO assay was performed with the lung tissues of the mice. Values are expressed as the mean ± SEM of 5 mice. Similar experiment was performed three times. ^*∗*^ *P* and ^*∗∗*^ *P* were less than 0.05, compared with the only LPS treated.

Next, we tested whether PIC II suppresses lung inflammation in LPS-induced ALI mouse model (Figure 4). C57BL/6 (*n* = 5/group) mice received either an intratracheal (i.t.) spraying of PBS (Figure 4(a)) or LPS (2 mg/kg body weight, Figures 4(b), 4(c), and 4(d)). At 2 h after the treatment, mice received additional i.t. spraying of PBS (Figures 4(a) and 4(b)) or PIC II in two different amounts (0.5 or 1 mg/kg body weight, Figures 4(c) and 4(d), resp.). At 24 h after LPS treatment, mice were sacrificed for the analysis of lung inflammation. Histologic analyses of lung tissue show that i.t. spraying of LPS induced robust lung inflammation featured by increased cellular infiltration and hyaline changes (Figure 4(b)), compared with PBS-treated mice (Figure 4(a)). However, i.t. spraying of PIC II suppressed lung inflammation (Figures 4(c) and 4(d)), in which 1 mg/kg of PIC II was more potent (Figure 4(d)). For the confirmation of lung inflammation suppressed by PIC II, BAL was performed and cells in the BAL fluid were counted. As shown in Figure 5(a), LPS treatment induced cellular infiltration to the lung, compared with PBS-treated control (1st and 2nd columns). Similar to Figure 4, however, PIC II treatment significantly reduced the cellular infiltration (2nd, 3rd, and 4th columns). Differential counting of cells in BAL fluid (Figure 5(b)) revealed that LPS treatment induced mostly neutrophil infiltration (2nd empty column), which was suppressed by PIC II treatment (3rd and 4th columns), suggesting that PIC II suppresses neutrophil infiltration in the LPS-induced ALI mouse model. Consistent with this, the activity of myeloperoxidase, a characteristic enzyme found in neutrophils, was increased after LPS treatment (1st and 2nd columns in Figure 5(c)), which was suppressed by PIC II treatment (3rd and 4th columns).

**Figure 6:**
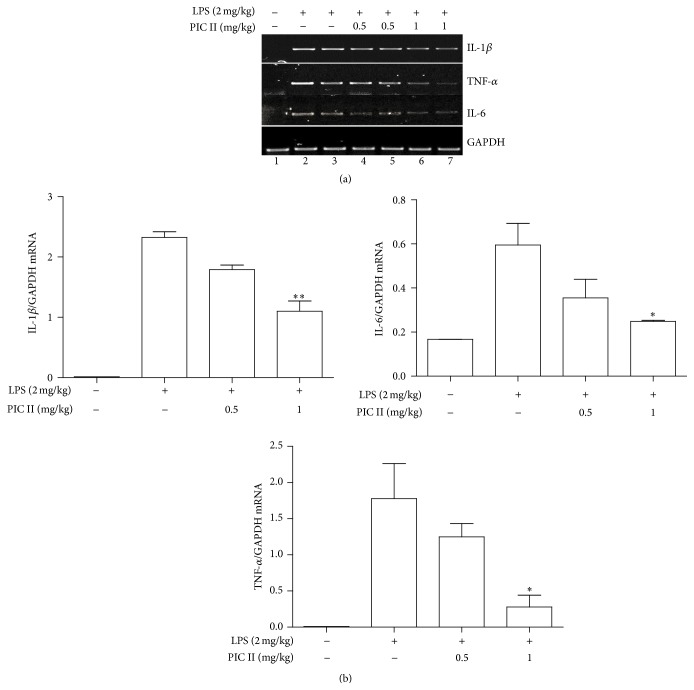
PIC II treatment decreases the expression of proinflammatory cytokine gene expression in the lungs of ALI mice. (a) Total RNA extracted from the lung tissues of ALI mice (*n* = 5) was analyzed by semiquantitative RT-PCR for the expression of representative proinflammatory cytokine genes. Two representatives in each group are shown. (b) Each band was quantitated by ImageJ, and relative expressions of those cytokine genes were calculated over a house-keeping gene, GAPDH. Data represent the mean ± SEM of each group in three measurements. ^*∗*^ *P* and ^*∗∗*^ *P* were less than 0.05 and 0.001, respectively, compared with the only LPS treated.

Since proinflammatory cytokines play a key role in the process of inflammation, we also examined whether PIC II affects the expression of those cytokines. Total RNA was extracted from the lung tissues of the mice treated as in Figure 4 and analyzed by semiquantitative RT-PCR for the expression of IL-1*β*, TNF-*α*, and IL-6. As shown in Figures 6(a) and 6(b), LPS treatment induced the expression of these cytokine genes, compared with the PBS-treated control (lanes 1, 2, and 3), which was suppressed by PCI II treatment (lanes 4 to 7). Taken together, these results show that PIC II suppressed neutrophilic lung inflammation elicited by LPS.

### 3.5. PIC II Induces the Phosphorylation of Smad 2 in Mouse Lungs

**Figure 7:**
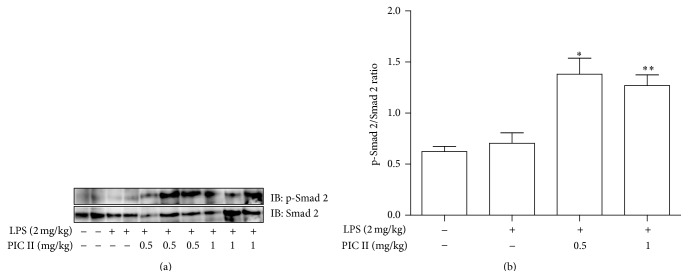
PIC treatment increased the level of phosphorylated form of Smad 2 in the lungs of ALI mice. (a) Lung tissues (2-3 lung tissues/group) were collected from mice treated as indicated. Total proteins were isolated from the lung tissues and analyzed for phosphorylated Smad 2 (p-Smad 2) by Western blot. The membrane was stripped and reprobed for Smad 2. (b) Each band was quantitated by ImageJ, and the levels of p-Smad 2 in each group were calculated over Smad 2. Data represent the mean ± SEM of three measurements. ^*∗*^ *P* and ^*∗∗*^ *P* were less than 0.05, compared with the untreated control.

Finally, we examined whether the suppressive effect of PIC II is associated with increased phosphorylation of Smad 2 in the lung. Total protein was extracted from the lung tissues of the mice treated as described in Figure 4 and analyzed by Western blotting for the phosphorylated form of Smad 2 over Smad 2. As shown in Figure 7(a), PIC II treatment induced phosphorylation of Smad 2, which was statistically significant (Figure 7(b)). Therefore, these results suggest that PIC II is able to activate TGF-*β* signaling.

## 4. Discussion

In this study, we examined how PIC II exerts its anti-inflammatory effect and whether PIC II is effective in suppressing neutrophilic lung inflammation using LPS-induced ALI mouse model. We found that PIC II induced the phosphorylation of Smad 2, a key transcription factor in TGF-*β* signaling, in RAW 264.7 cells and in mouse lung tissues, and we also found that PIC II was effective in suppressing neutrophilic lung inflammation in the mouse model used in this study. Based on this experimental evidence, we suggest that PIC II suppresses neutrophilic lung inflammation which may be associated with Smad 2 phosphorylation.

PIC II is known as a major constituent found in the dried rhizomes of* P. scrophulariiflora* that has long been used to treat various inflammatory diseases as part of traditional Asian medicine [24]. Like many herbal medicines in traditional medicine, however, the usage of this herb is limited because the mechanism by which* P. scrophulariiflora* exerts its alleged effects has been elusive. Although incomplete to understand the precise mechanism for the anti-inflammation effect of* P. scrophulariiflora*, our results suggest that* P. scrophulariiflora* exerts its anti-inflammatory effect via PIC II. Because of our results which demonstrate that the possible anti-inflammatory activity of PIC II may be associated with TGF-*β* signaling, our study offers a glimpse at one of the possible mechanisms by which* P. scrophulariiflora* executes its pharmacologic effect.

Inflammation is a key underlying pathophysiologic process in inflammatory lung diseases including ALI and chronic obstructive pulmonary disease (COPD) [29]. In ALI, bacterial infection is a major cause of the disease [2, 4, 30]. Macrophages resident in the lung play a key role in initiating inflammation by sensing invading bacteria via TLRs and other receptors that recognize pathogen-associated molecular patterns (PAMPs) of the pathogens [31]. Engagement of these receptors with bacterial ligands results in activation of NF-*κ*B [32]. Activated NF-*κ*B induces various proinflammatory cytokines that attract various inflammatory cells including neutrophils in blood to the lung, thereby exacerbating inflammatory responses to a severe lung inflammation [33]. Therefore, regulation of NF-*κ*B activity can be one of the steps to dampen inflammatory response. During our study, it was reported that PIC II downregulates the expression of TLR4 and NF-*κ*B, suggesting that the anti-inflammatory effect of PIC II is mediated by suppressing NF-*κ*B [34]. Therefore, we expected a similar result in our study. However, in our experimental setting, we could not detect NF-*κ*B activity significantly suppressed by PIC II. While this discrepancy may stem from differences in experimental settings and target diseases, our results cannot exclude the possibility that PIC II suppresses NF-*κ*B activated via receptors other than TLR4 because we only measured the effect of PIC II on NF-*κ*B activated through TLR4 but not via other receptors, for example, TNF-*α* and IL-1*β*, which also activate NF-*κ*B [25].

TGF-*β* signaling affects various biological processes, including tissue growth and cell death, although the ramifications of TGF-*β* signaling are dependent on cell types [35]. In the pathophysiology of inflammatory diseases, TGF-*β* signaling contributes to the suppression of inflammation [36] and plays an important role in regulating ALI [37]. Although there are several conflicting results on the role of TGF-*β* in regulating neutrophils [38, 39], the consensus is that TGF-*β* plays a key role in resolving neutrophilic lung inflammation. In neutrophilic lung inflammation, which is notable in ALI, TGF-*β* enhances resolution of inflammation in ALI mice through regulatory T cells (Foxp3^+^ CD4^+^ CD25^+^ Treg cells) [40] and IL-6 [41]. Mechanistically, the outcome of TGF-*β* signaling hinges on Smad proteins and cofactors recruited by Smad proteins at the level of transcription regulation in a cell [36]. Prototypic TGF-*β* signaling involves Smad 2/3; TGF-*β* binding to its cognate receptor phosphorylates Smad 2/3 and phosphorylated Smad 2/3 moves to the nucleus, forming a complex with coactivator Smad 4 to induce the expression of TGF-*β* dependent genes [35]. Our result shows that PIC II treatment induced the phosphorylation of Smad 2 in RAW 264.7 cells. However, it appears that the phosphorylation of Smad 2 elicited by PIC II was insufficient to induce SBE-driven luciferase activity because PIC II increased the SBE-driven luciferase activity only in the presence of a constitutively active form of TGF-*β*R but not PIC II alone. Therefore, it is likely that although PIC II can phosphorylate Smad 2, it requires other signaling events to fully activate TGF-*β* signaling. Therefore, it is possible that PIC II helps in enhancing or facilitating TGF-*β* signaling cascade by further phosphorylating Smad 2. Nevertheless, in the ALI mice, PIC II suppressed neutrophilic lung inflammation with concomitant decrease of inflammatory cytokine gene expressions. Furthermore, PIC II induced the phosphorylation of Smad 2 in lung tissues. Therefore, these results suggest that PIC II suppresses neutrophilic lung inflammation in ALI, which is associated, at least in part, with TGF-*β* signaling.

## 5. Conclusion

In this study, we examined whether PIC II has an anti-inflammatory activity and is applicable for treating ALI. We found that PIC II was effective in suppressing neutrophilic lung inflammation in LPS-induced ALI mouse model, the effect of which was associated at least in part with TGF-*β* signaling. Our results suggest that PIC II plays a role as an anti-inflammatory constituent in* P. scrophulariiflora*.

## Figures and Tables

**Table 1:** Oligonucleotide primers used for RT-PCR in this study.

Target gene	Oligonucleotide sequences (5′ to 3′ direction)	Expected size
IL-6	CTG GTG ACA ACC ACG GCC TTC A ATG CTT AGG CAT AAC GCA CTA GGT	600 bp

IL-1*β*	TCA TGG GAT GAT GAT GAT AAC CTG CT CCC ATA CTT TAG GAA GAC ACG GAT T	502 bp

TNF-*α*	GGC AGG TCT ACT TTG GAG TCA TTG C ACA TTC GAG GCT CCA GTG AAT TCG G	307 bp

GAPDH	GGA GCC AAA AGG GTC ATC AT GTG ATG GCA TGG ACT GTG GT	203 bp

## Abbreviations

ALI: Acute lung injury
PIC II: Picroside II
Nrf2: Nuclear erythroid 2-related factor 2
Smad 2: SMA (small body size) and MAD (mothers against decapentaplegic) 2
TGF-*β*: Transforming growth factor-*β*
SBE: Smad-binding element
LPS: Lipopolysaccharide.
